# Mistrust surrounding vaccination recommendations by the Japanese government: results from a national survey of working-age individuals

**DOI:** 10.1186/s12889-015-1772-8

**Published:** 2015-04-26

**Authors:** Koji Wada, Derek R Smith

**Affiliations:** International Health Cooperation, National Center for Global Health and Medicine, 1-21-1 Toyama Shinjuku-ku, Tokyo, Japan; School of Health Sciences, Faculty of Health and Medicine, University of Newcastle, Ourimbah, Australia

**Keywords:** Vaccination, Immunisation, Mistrust, Information, General health conditions, Population

## Abstract

**Background:**

Considering that public attitudes on vaccine safety and effectiveness are known to influence the success of vaccination campaigns, an increased understanding of socio-demographic characteristics might help improve future communication strategies and lead to greater rates of vaccination uptake. This study investigated associations between mistrust for governmental vaccine recommendations and the socio-demographic characteristics of working-age individuals in Japan.

**Methods:**

A web-based, cross-sectional survey of vaccination attitudes was conducted among 3140 Japanese people aged 20 to 69 years. Multiple logistic regression analysis was used to examine statistical associations between vaccination attitudes and socio-demographic characteristics, including the participant’s most trusted information resources, demographic factors and general health conditions.

**Results:**

A total of 893 (28.4%) individuals reported a general mistrust towards the Japanese government’s recommendations for vaccination. Respondents who did not trust official government sources were more likely to consider friends, the internet and books (for both genders); family members and newspapers (among women only); and television (among men only), as the most trusted resources for vaccination-related information. Relatively poor health in men was associated with a general mistrust of vaccination recommendations (adjusted Odds Ratio (aOR): 1.37, 95% Confidence Interval (95% CI): 1.07-1.69). A trend towards worsening general health was also associated with decreasing trust in vaccination recommendations by female respondents as follows: those reporting relatively good health (aOR: 1.24, 95% CI: 1.02-1.47); relatively poor health (aOR: 1.55, 95% CI: 1.22-1.90); and poor health (aOR: 2.10, 95% CI: 1.41-2.63) (p for trend < 0.05).

**Conclusions:**

Overall, this study suggests that communication strategies for rebuilding public trust in vaccination safety need to be urgently addressed in Japan. Such protocols must consider the information sources that working-age populations are most likely to utilize in this country, as well as their general health conditions, especially among females.

## Background

It is well-known that community and individual attitudes towards governmental recommendations can influence the ultimate success of vaccination campaigns [1]. During the influenza A (H1N1) pandemic of 2009 for example, many countries conducted campaigns for promoting vaccination [2,3]. The overall proportion who were vaccinated remained low however, even among health care workers, partly due to a perceived risk of side effects [4,5]. In Japan for example, only around one-quarter of the working-age population chose to be vaccinated against influenza [6]. For females aged 60–69 years who were recommended by the government to be vaccinated against influenza, almost one-third simply refused because of a perceived lack of confidence in the effectiveness of influenza vaccinations [7]. Even though all governmental recommendations for vaccination are based on solid scientific evidence methodically collected over a number of years, there still remains a certain degree of mistrust by the general public regarding vaccination efficacy and the perceived risk of side effects [8].

It is possible that individuals may not trust the Japanese government’s recommendations on vaccination simply due to confusing information from different sources. In June 2013, for example, *Human papilloma virus* (HPV) vaccination (which had been recommended as a routine vaccination in Japan since April of that year), was officially suspended based on recommendations by a scientific committee organized by the Japanese government. This reversal occurred due to a complex regional pain syndrome being reported by some individuals who had received HPV vaccination [9]. Although the Japanese government’s urgent response (despite insufficient evidence of a causal relationship) was to ensure population safety, such decisions can lead to confusion among the general public, and even lead to mistrust of the government’s overall recommendations for vaccination. Not all countries manage adverse vaccine events in this manner, however. In the United States, for example, immunization recommendations are made by an independent advisory committee comprising multiple stakeholders, such as the Advisory Committee on Immunization Practices, rather than a committee organized by the government, as happened in the recent Japanese case [10]. Therefore, the decision-making systems, which ensure people’s trust in vaccination recommendations in Japan, may need to be reviewed [11].

Mistrust for these recommendations may also result from alarmist news reports suggesting adverse vaccination-related events, as well as a certain degree of fluctuation in the overall government recommendations [9]. A previous Japanese study, for example, reported that women aged 40–60 years tended to avoid influenza vaccination due to a fear of potential side effects [7]. This particular generation was likely influenced by stories in the mass-media and elsewhere regarding potential side effects and lawsuits for compensation at the time that their children were being vaccinated [12].

Having a better understanding of the socio-demographic characteristics associated with mistrust towards governmental recommendations might therefore, offer a way forwards to increased vaccination coverage in Japan, as elsewhere. Indeed, some studies have already shown that the perception of vaccine safety can relate to demographic and social factors such as age, information sources and education levels [5,13]. As such, the current study was undertaken to investigate associations between mistrust for governmental recommendations on vaccination and social background in the working-age population of Japan.

## Methods

### Data collection

A total of 3000 Japanese individuals aged 20 to 69 years were recruited from those who had previously registered with an online survey company. Registrants were people interested in voluntarily participating in a survey that provided financial incentives. Using a random number generator, the survey company selected individuals from the database and invited them to participate in the study during January 2014. Recruitment was originally intended to cease when the total number of participants had reached the target of 3,000. However, 3,140 persons eventually agreed to participate from a total of 7,087 individuals who were initially contacted, from a grand total of 1.60 million registrants. Participants were classified into five age groups (20–29, 30–39, 40–49, 50–59, and 60–69 years), and by gender, since the distribution of registrants for the internet survey might be expected to have been biased. Our sample size was calculated based on the expected percentage who would mistrust the Japanese government’s policy on vaccination (30%), with an expected precision of ±5%. The required sample size was calculated at 264 individuals for each age range category and gender. As a result, we aimed to recruit 300 individuals for each age group and gender for the current study.

### Questionnaire

Questions included basic demographic information such as age, sex, and completed education levels (high school or lower, college or vocational school, and, university education or higher); the most trusted information source for determining whether to have vaccinations, and self-rated general health conditions. The Japanese questionnaire was translated into English by a professional bilingual translator and then back-translated into Japanese by another bilingual translator to check against the original. To determine their perception of vaccination recommendations by the Japanese government, the question asked was: “Do you trust the recommendations by the government about vaccination?” with possible answers of: “1 = yes, certainly”, “2 = mostly”, “3 = not very much”, or “4 = no I don’t”. To determine their most trusted information source regarding whether to have vaccinations, we asked: “Which information source do you trust the most when deciding whether to get vaccinated? (select one only)”, with the possible answers of: “healthcare providers, such as doctors and nurses”, “public administration of the national or local government”, “family”, “friends”, “TV”, “newspapers”, “the Internet”, “books”, and “none of the above”. We also enquired about the participant’s general health condition with the following question: “Regarding your current health status, please choose the most applicable answer from the following options (select one only): “I am in good health”, “I am in relatively good health”, “I am in relatively poor health”, and “I am in poor health”. Current smoking status was also requested, using the following options: “I currently smoke”, “I have quit”, and “I have never smoked”.

### Statistical analysis

Univariate statistical analysis, chi-square comparison and logistic regression was undertaken to examine potential associations between mistrust of the governmental recommendations on vaccination, and the aforementioned demographic variables. Logistic regression was used to examine potential associations between demographic variables and the outcome of interest (that being, mistrust of the Japanese government’s recommendations on vaccination). Statistical analysis was also undertaken by gender, to help establish more detailed background factors. Responses on mistrust for the government’s recommendations on vaccination were initially measured on a four-point scale (“1 = yes, certainly”, “2 = mostly”, “3 = not very much”, or “4 = no I don’t”); and then collapsed into a two-point scale, as follows: (1: Mistrust=”not very much” and “no I don’t”; and 0: Trust=”yes, certainly” and “mostly”). A further adjustment was made for age and highest completed education levels. Trend analysis for the odds ratios of general health conditions for women was conducted. All analyses were performed using IBM SPSS Statistics 20, with the statistical significance set at p < 0.05. We hypothesized that gender in the Japanese working demographic might be a factor in this equation, particularly relating to the most trusted information sources; and as a result, we analyzed the data by gender. Odds ratios were adjusted using Zhang’s correction formula for common outcomes, given that the prevalence of mistrust was relatively high [14].

### Ethics statement

This study was approved by the Human Ethics Committee of the National Center for Global Health and Medicine in Japan. Only those who agreed to participate in this study were able to access and then answer questions on the website.

## Results

A total of 3140 persons, including 1571 men and 1569 women, participated in this study. Demographic characteristics of the participants are shown in Table 1. By proportion, the most trusted information resource for determining whether to be vaccinated was health care workers (44.1%). With regard to general health conditions, ‘relatively good health’ was the most common status reported by participants (54.9%). Overall, 28.4% of participants expressed mistrust towards the Japanese government’s official recommendations on vaccination.

**Table 1:** **Mistrust of the government’s vaccination recommendations and demographic variables in the working-age population of Japan (N=3,140)**

	**n**	**(%)**
**Age range (years)**		
20-29	630	(20.1)
30-39	627	(20.0)
40-49	632	(20.1)
50-59	626	(19.9)
60-69	625	(19.9)
**Gender**		
Male	1,571	(50.0)
Female	1,569	(50.0)
**Most trusted information source on vaccination**		
Health care workers	1,385	(44.1)
Information from the government	285	(9.1)
Family	267	(8.5)
Friends	50	(1.6)
Television	180	(5.7)
Newspapers	91	(2.9)
The Internet	78	(2.5)
Books	30	(1.0)
Nothing in particular	774	(24.6)
**General health status**		
Good health	874	(27.8)
Relatively good health	1,724	(54.9)
Relatively poor health	424	(13.5)
Poor health	118	(3.8)
**Smoking status**		
Never smoked	682	(21.7)
Ex-Smoker	1,869	(59.5)
Current smoker	589	(18.8)
**Education level**		
High school or lower	945	(30.1)
College or vocational school	792	(25.2)
University level or higher	1,403	(44.7)
**Do you trust the government’s recommendations about vaccination?**		
No (Mistrust)	145	(4.6)
Not very much (Mistrust)	748	(23.8)
Mostly (Trust)	2,061	(65.6)
Yes, certainly (Trust)	186	(5.9)

Table 2 indicates statistical associations between mistrust for government recommendations on vaccination and demographic variables. A relatively high proportion of those who reported not trusting government recommendations on vaccination reported that their main information source were books, even though this option comprised a relatively small proportion of the total population under study. According to chi-square analysis, using the television as the most trusted information source and never having smoked displayed significant differences by gender (p < 0.05).

**Table 2:** **Association of mistrust regarding government vaccination recommendation and the studied variables in Japan (N=3140)**

	**Male**				**Female**			
	**Mistrust**		**Trust**		**Mistrust**		**Trust**	
	**(n=464)**		**(n=1107)**		**(n=429)**		**(n=1140)**	
	**n**	**(%)**	**n**	**(%)**	**n**	**(%)**	**n**	**(%)**
**Most trusted information source on vaccination**								
Health care workers	138	(20.8)	527	(79.2)	126	(17.5)	594	(82.5)
Information from the government	10	(6.2)	152	(93.8)	8	(6.5)	115	(93.5)
Family	32	(24.4)	99	(75.6)	40	(29.4)	96	(70.6)
Friends	10	(45.5)	12	(54.5)	10	(35.7)	18	(64.3)
Television	27	(30.3)	62	(69.7)	15	(16.5)	76	(83.5)
Newspapers	11	(26.2)	31	(73.8)	15	(30.6)	34	(69.4)
The Internet	15	(37.5)	25	(62.5)	18	(47.4)	20	(52.6)
Books	9	(64.3)	5	(35.7)	11	(68.8)	5	(31.2)
None of the above	212	(52.2)	194	(47.8)	186	(50.5)	182	(49.5)
**General health status**								
Good health	100	(25.9)	286	(74.1)	109	(22.3)	379	(77.7)
Relatively good health	248	(28.3)	627	(71.7)	229	(27.0)	620	(73.0)
Relatively poor health	91	(38.2)	147	(61.8)	70	(37.6)	116	(62.4)
Poor health	25	(34.7)	47	(65.3)	21	(45.7)	25	(54.3)
**Smoking status**								
Never smoked	212	(29.8)	500	(70.2)	302	(26.1)	855	(73.9)
Ex-Smoker	117	(27.4)	310	(72.6)	71	(27.8)	184	(72.2)
Current smoker	135	(31.2)	297	(68.8)	56	(35.7)	101	(64.3)

Table 3 indicates the results of multiple logistic regression analysis. Respondents who reported mistrust for vaccination were less likely to consider information from the government as their most trusted information source on vaccination, as follows: among men (adjusted Odds Ratio (aOR): 0.33; 95% Confidence Interval (CI): 0.18-0.59), and women (aOR: 0.39; 95% CI: 0.20-0.74) compared with participants who did trust government vaccination. Respondents who did not trust vaccination recommendations were more likely to consider other information sources as being trustworthy, as follows: the Internet (men, aOR: 1.67; 95% CI: 1.12-2.22; women, aOR: 2.19; 95% CI: 1.58-2.73); books (men, aOR: 2.53; 95% CI: 1.67-3.05; women, aOR: 2.99; 95% CI: 2.19-3.40); newspapers (women, aOR: 1.56; 95% CI: 1.03-2.15), family (women, aOR: 1.60; 95% CI: 1.23-1.99); and friends (men, aOR: 1.96; 95% CI: 1.24-2.60; women, aOR: 1.80: 95% CI: 1.11-2.51). Among female respondents, those reporting relatively good health (aOR: 1.24; 95% CI: 1.02-1.47), relatively poor health (aOR: 1.55; 95% CI: 1.22-1.90), and poor health (aOR: 2.10; 95% CI: 1.41-2.63) had a significantly higher likelihood of mistrust for vaccination (p for trend < 0.05). Being a current smoker was weakly associated with a mistrust of vaccination among men (aOR: 1.31; 95% CI: 0.97-1.74). Age and differences in education levels were not significantly associated with vaccination mistrust during multiple regression analysis.

**Table 3:** **Statistical associations with mistrust attitudes regarding government vaccination information in Japan (N=3140)**

	**Male**				**Female**			
	**Crude**		**Multivariate**		**Crude**		**Multivariate**	
	**aOR**	**(95% CI)**	**aOR**	**(95% CI)**	**aOR**	**(95% CI)**	**aOR**	**(95% CI)**
**Most trusted information source on vaccination**								
Health care workers	1.00	-	1.00	-	1.00	-	1.00	-
Information from the government	0.32	(0.17-0.58)*	0.33	(0.18-0.59)*	0.40	(0.20-0.75)*	0.39	(0.20-0.74)*
Family	1.15	(0.85-1.51)	1.18	(0.87-1.55)	1.55	(1.20-1.93)*	1.60	(1.23-1.99)*
Friends	1.94	(1.22-2.57)*	1.96	(1.24-2.60)*	1.80	(1.13-2.51)*	1.80	(1.11-2.51)*
Television	1.39	(1.01-1.80)*	1.36	(0.99-1.78)	0.95	(0.60-1.41)	0.91	(0.56-1.37)
Newspapers	1.23	(0.74-1.82)	1.23	(0.74-1.83)	1.61	(1.07-2.18)*	1.56	(1.03-2.15)*
The Internet	1.66	(1.12-2.21)*	1.67	(1.12-2.22)*	2.25	(1.65-2.77)*	2.19	(1.58-2.73)*
Books	2.52	(1.65-3.04)*	2.53	(1.67-3.05)*	2.91	(2.09-3.37)*	2.99	(2.19-3.40)*
None of the above	2.16	(1.94-2.36)*	2.14	(1.92-2.35)*	2.36	(2.12-2.58)*	2.38	(2.13-2.60)*
**General health condition**								
Good health	1.00	-	1.00	-	1.00	-	1.00	-
Relatively good health	1.09	(0.90-1.31)	1.09	(0.89-1.32)	1.19	(1.02-1.38)*	1.24	(1.02-1.47)*
Relatively poor health	1.46	(1.17-1.76)*	1.37	(1.07-1.69)*	1.60	(1.29-1.93)*	1.55	(1.22-1.90)*
Poor health	1.33	(0.92-1.79)	1.24	(0.82-1.74)	1.90	(1.36-2.42)*	2.10	(1.41-2.63)*
**Smoking status**								
Never smoked	1.00	-	1.00	-	1.00	-	1.00	-
Ex-Smoker	0.97	(0.75-1.24)	0.94	(0.71-1.22)	0.95	(0.76-1.12)	0.93	(0.71-1.19)
Current smoker	1.45	(1.11-1.86)*	1.31	(0.97-1.74)	1.02	(0.82-1.26)	0.96	(0.75-1.22)

aOR: adjusted Odds Ratio by Zhang’s formula; CI: Confidence Interval; *p<0.05.

With regard to educational level among females, univariate logistic regression suggested that having a university level or higher education was important when compared to those with high school or lower education (aOR: 0.77; 95% CI: 0.60-0.99); although this relationship disappeared when an adjusted model was used (aOR: 0.92; 95% CI: 0.70-1.20). For males, educational level was not associated with mistrust of the government’s vaccination recommendation during regression analysis.

## Discussion

This study provides an important insight into public attitudes towards official Japanese government vaccination recommendations for one of the first times. Our investigation has also demonstrated some potential associations between the most trusted information sources for vaccination and the general health status of the working-age population in this country. Among them, approximately one-quarter of respondents stated that they did not trust the Japanese government’s recommendations on vaccination. The relationship between mistrust and subsequent vaccination status are multifactorial and complicated however, and it is important to note that mistrust against governmental recommendations does not necessarily lead to a lack of vaccine uptake.

Health care workers are well-recognized as a trusted information source in many studies [8], and this was also observed in the current investigation, with approximately half of all participants answering that health care workers were their most trusted information source. Nevertheless, about one-fifth of the respondents still harbored some mistrust towards the government’s recommendations. As such, Japanese health care workers should take this into consideration when supporting patient decisions regarding vaccination when based on the government’s recommendation [15]. Although a previous study reported that individuals can make distinctions between the advice given by doctors from different settings with regard to vaccination [16], this option may not be as likely among busy people who occupy the working demographic. This suggests that educational opportunities which involve health care workers reaching out to working populations might need to collaborate more effectively with the mass media, for example. Educational interventions to increase vaccine uptake might also consider targeting working populations directly at their place of employment [17].

Traditional mass media sources including newspapers and television, represent an important tool for distributing information on vaccination and in recent years the broader availability of the internet has also influenced the way in which information is distributed [18,19]. Due to a lack of regulation, however, the mass media (particularly the internet) makes it easy to promote topics which people simply feel strongly about, such as the fear of potentially negative events; rather than presenting a balanced interpretation of the facts by recognized medical experts. On one hand, the perception of ‘risk’ is in itself, an often difficult concept for people to fully appreciate [20]. On the other, it is well-known that the internet (including social networking sites), can be a source of negative or incorrect information on vaccinations [21]. This hypothesis is supported by the current study where participants who considered the Internet their most trusted information resource for making a decision on vaccination also mistrusted government recommendations on the same topic.

At a personal level, individuals are known to seek information on their beliefs within their social context [8,21]. Survey participants who responded that books (which would clearly need to be purchased or borrowed from a library) are their most trusted information source may be more enthusiastic collectors of information based on their social context. A database search of Japanese books [22] revealed that there were 77 different titles on vaccination, of which 40 were specifically aimed at health care workers. The remaining 37 books were for the general public, and a basic classification into positive or negative aspects revealed that 73% focused on the negative aspects of vaccination, with some even describing conspiracy theories regarding vaccination [23]. It is reasonable to suspect that individuals who seek vaccination-related information from books may already harbor negative perceptions towards vaccination. Even though the proportion of such individuals in the current study who chose books as the most trusted information source for vaccination was small (less than 1%), their total number may still be sufficient to spread negative information regarding vaccination in Japan, especially via the internet. Given that controlling the internet and mass media is not immediately feasible, additional strategic efforts to promote positive messages regarding vaccination are clearly needed in Japan. One way forwards might be to more effectively partner with the community to disseminate the undoubted beneficial aspects of vaccination, while simultaneously tackling incorrect perceptions and providing scientifically-sound information from trusted information sources [24].

Our current study demonstrated that among working-age populations in Japan, family and friends may represent important negative sources when making vaccination-related decisions. Recently it has been suggested that ‘network phenomena’ are relevant to individual lifestyles, such as eating habits and smoking [25,26], and this may also be the case for vaccination [27]. Given that network phenomena might also help spread positive health behavior (such as promoting vaccination) [28], new strategies that incorporate social networking to change peoples’ behavior on vaccination are anticipated in further research [29]. On the other hand, a previous study revealed that family and friends’ recommendation was a positive factor in influenza vaccination, especially among those aged in their 20s [7]. Further research is therefore needed to help better understand the effects of networking behavior on vaccination uptake by age group.

Interestingly, our study showed that worsening self-rated health conditions for women were significantly associated with mistrust for the governmental recommendations on vaccination. During the influenza A (H1N1) pandemic of 2009, adults at risk of medical complications such as asthma, diabetes and obesity, were recommended to be vaccinated [30,31]. While this is clearly sound medical advice, it may not be viewed as such by at-risk individuals with pre-existing concerns, who would then be unwilling to be vaccinated. It is reasonable to assume that individuals in poor health may fear adverse side effects to a heightened degree and thus, be more concerned with vaccine safety [8]. Detailed communication strategies for these people are therefore necessary to improve the success of future vaccination campaigns.

It is well-known that smokers tend to have poorer health behaviors than their non-smoking counterparts [32] and obtaining vaccinations is not an exception to this rule [6,33]. In the current study we found a weak association between mistrust for governmental recommendations for vaccination in Japan and individual smoking status. As such, more effective interventions to help smokers obtain vaccinations should be addressed during vaccination campaigns; especially in those such as pandemic flu, where smokers can be at a higher risk of developing complications [34]. The workplace is known to offer important opportunities for public health interventions, including tobacco control; [35] and given that the population in the current study consisted of working-age persons, may offer an ideal way forwards in this regard [36].

Education levels might also reflect access to information and decision making processes regarding topics such as vaccination. At least one previous study [13] has shown that individuals with a bachelor degree or higher were more likely to believe in the safety of influenza vaccination compared with those having lower levels of education. Our current study also revealed that only female participants who had completed university education or higher had a lower risk of mistrust for governmental recommendations on vaccination during univariate statistical analysis, but not during multivariate analysis. Further research is therefore needed to clarify whether this represents a statistical anomaly or a genuine finding.

As with any population-based survey, the current study may have included various inherent limitations. First, the population studied was recruited through an online survey company and therefore, they must have been able to access a computer and be able to use the internet. Such a population may not be representative of all Japanese people. Second, we were unable to establish why some individuals refused to participate in the survey, and this may have affected the outcome. Third, the current investigation was cross-sectional in design and was therefore unable to confirm any causal relationships. Fourth, at its core, the measuring of mistrust towards vaccination was undertaken by using a single question, since an appropriately validated questionnaire on this topic could not be found during the study design phase. Finally, it should also be noted that some of the health-related information was subjective in nature. As such, future research on this topic might consider developing a standardized international questionnaire targeting vaccination mistrust. Further studies are also needed to establish whether people are actually satisfied with the information they receive on vaccination [37], given that they might simply mistrust governmental recommendations due to a lack of information. Furthermore, additional research should also be conducted to establish whether population-based mistrust for vaccination recommendations is specific for adult or child vaccinations, or both.

## Conclusion

Overall, this study suggests that communication strategies for rebuilding trust in governmental recommendations on vaccination should be addressed by considering the information sources that Japanese people trust, as well as their general health conditions; especially among females.
